# Shared HLA‐E and Mamu‐E Peptide Repertoires With Subtle Peptide Binding Differences Revealed by Combined nDSF‐ and Fluorescence Polarisation‐Based Methods

**DOI:** 10.1002/eji.70125

**Published:** 2026-01-16

**Authors:** Max N. Quastel, Sashini A. Ranawana, Bas W. A. Peeters, Andy van Hateren, Andrew J. McMichael, Geraldine M. Gillespie

**Affiliations:** ^1^ Nuffield Department of Medicine Centre for Immuno‐Oncology University of Oxford Oxford UK; ^2^ Institute For Life Sciences and Centre for Cancer Immunology Faculty of Medicine University of Southampton Southampton UK

**Keywords:** fluorescence polarisation, HLA‐E, Mamu‐E, MHC‐E, nano‐differential scanning fluorimetry, peptide binding

## Abstract

The primary function of MHC‐E—human leukocyte antigen (HLA)‐E in humans and Mamu‐E in rhesus macaques—relates to immune surveillance via CD94/NKG2x receptors expressed on NK cells. However, a secondary role where MHC‐E presents immunogenic peptides to CD8^+^ T cells that provide protective immunity in specific settings has also been described. Given the high sequence homologies between HLA‐E and Mamu‐E molecules, peptide binding similarities are assumed but not systematically explored, with most studies prioritising HLA‐E. Here, we have optimised and developed two complementary techniques to explore the peptide repertoires of specific HLA‐E and Mamu‐E subtypes. We established a label‐free, high‐throughput nano‐differential scanning fluorimetry (nDSF)‐based method, where peptide binding strength is measured through thermal stability (T_m_). This method revealed shared repertoires with occasional subtype‐specific peptide binding hierarchies for the MHC‐E types studied here, HLA‐E*01:03 and Mamu‐E*02:04. When combined with a fluorescence polarisation (FP) peptide competition assay, we show that half maximal inhibitory concentrations (IC_50_) correlate exponentially with nDSF‐acquired T_m_ data, revealing that modest T_m_ increments equate to marked IC_50_ differences, and hence substantial differences in relative peptide binding strengths. Collectively, these methodologies offer high‐throughput, scalable approaches to provide detailed peptide binding information for rhesus and human MHC‐E types.

## Graphical abstract

The MHC‐E peptide binding methods described here—nano‐differential scanning fluorimetry and fluorescence polarisation—demonstrate shared peptide binding repertoires with subtle binding hierarchy differences for the tested HLA‐E and Mamu‐E allotypes. When combined, these methods illustrate how minor MHC‐E‐peptide thermal stability changes equate to significant binding strength differences.

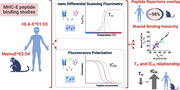

**Abbreviations**
FP: fluorescence polarisation
HLA‐E: human leukocyte antigen‐E
IC_50_
: half‐maximal inhibitory concentration
Mamu‐E: major histocompatibility complex E, rhesus macaques
MHC‐E: major histocompatibility complex E
nDSF: nano‐differential scanning fluorimetry
PBG: peptide binding groove
T_m_
: thermal stability

## 1 Introduction

Major histocompatibility complex E (MHC‐E)—human leukocyte antigen E (HLA‐E) in humans and Mamu‐E in rhesus macaques—represent a group of non‐classical MHC class Ib molecules with a primary function relating to natural killer (NK) cell‐mediated immune surveillance. In humans, HLA‐E typically presents the HLA class Ia and HLA‐G leader sequence‐derived nonamer VL9 (VMAPRT(L/V)(V/L/I/F)L), which is recognised by CD94/NKG2x receptors on NK cells and certain CD8^+^ T cells [1, 2, 3, 4, 5]. Interactions between the HLA‐E VL9 complex and NKG2A receptor exert an inhibitory effect on NK cells, and thus, when VL9 peptide supply is limited, such as in viral infection, where HLA class I is downregulated, this loss of signal releases NK cells from inhibition [5]. HLA‐E VL9 surface presentation consequently reflects the functional status of the cellular MHC class I peptide presentation system, allowing NK cells to assess cellular health. Recent research, however, has shown that HLA‐E can also present pathogen‐derived peptides to CD8^+^ T cells [6, 7, 8, 9, 10, 11]. Although many of these peptides bind weakly to HLA‐E through partial or suboptimal peptide binding pocket occupancy, they have been reported to elicit T cell responses in certain intracellular bacterial and viral infections, evidenced through tetramer staining [7, 11] and via T cell proliferation and cytotoxicity assays [7, 8, 9, 12].

Much of the renewed interest in MHC‐E and adaptive immunity stems from a RhCMV vectored SIV vaccine, where the elicited Mamu‐E restricted CD8^+^ T cell responses to SIV‐derived peptides protected SIV challenge in approximately 55% of rhesus macaques [13, 14]. If a similar protection could be replicated for HLA‐E in humans, this offers a novel pathway for vaccine strategies that target HIV and other diseases. Compared with HLA‐E, Mamu‐E is more polymorphic, with 33 alleles currently reported [15]. However, the peptide binding groove (PBG) sequences of these Mamu‐E subtypes are highly conserved, with most polymorphisms located outside this region [15]. HLA‐E, in contrast, carries only two common alleles across the human population—HLA‐E*01:01 and HLA‐E*01:03 [16] ‐ that differ by a single amino acid (arginine or glycine, respectively) at position 107 outside the PBG [17]. Therefore, the limited genetic polymorphism of HLA‐E [17, 18], its broad expression across most cell and tissue types [19], and its resistance to downregulation in cancer and infectious disease settings [20, 21, 22, 23, 24] make it a promising platform as a near “universal” immune target.

HLA‐E and Mamu‐E peptide binding has previously been explored [11, 13, 25, 26], but to a lesser extent for Mamu‐E. Various peptide binding assays have been developed, including a single‐chain trimer (SCT) cell‐surface expression assay, peptide‐exchange sandwich ELISAs, and HLA‐E surface stabilisation assays performed by peptide pulsing experiments [11, 13, 25, 26, 27]. The SCT assay is a cell‐based method in which a DNA expression vector encoding a test peptide linked to β2‐microglobulin (β2m) and the HLA‐E or Mamu‐E heavy chain is transfected into HEK293T cells, following which cell surface expression is measured as a proxy of peptide binding using antibodies specific for HLA‐E (3D12) and Mamu‐E (4D12) [13, 27, 28]. The peptide‐exchange sandwich ELISA samples complexes of HLA‐E refolded with a UV‐sensitive VL9 peptide, which, when either exposed to UV light or incubated overnight, disassociates, but can be rescued in the presence of excess binding peptide [25]. The sandwich ELISA assay, based on heavy chain capture (using 3D12 antibody) and β2m detection, serves as a measure of peptide binding strength [25]. Although both methods provide very reliable and quantitative measures of peptide binding, there are some drawbacks, as both assays are relatively low‐throughput and do not provide nuanced measurements that enable accurate quantification of the scale of relative peptide binding strength differences.

More recently, we have quantified peptide binding to HLA‐E through the measurement of changes in the thermal stability (T_m_) of HLA‐E peptide complexes using nano‐differential scanning fluorimetry (nDSF) [11, 29, 30]. nDSF is a technique which measures protein unfolding through monitoring fluorescence changes as a temperature gradient is applied to samples. Specifically, this label‐free method [31] measures fluorescence changes affecting tryptophan residues (and to a lesser extent tyrosine), which become more solvent exposed as the protein unfolds with increasing temperature, resulting in a fluorescence peak shift from 330 to 350 nm. Plotting the first derivative of the 350 nm/330 nm ratio—the point at which this curve peaks—corresponds to the temperature at which 50% of the protein is unfolded and the thermal stability (T_m_) of the protein complex. Compared with the assays discussed above, nDSF offers a higher‐throughput platform and takes minimal time to set up and run.

Fluorescence polarisation (FP), also known as fluorescence anisotropy, represents a separate technique that has been successfully employed to interrogate peptide binding to MHC class Ia types [32]. Here, polarised light is used to excite a fluorescently labelled peptide, and the polarisation of the emitted light from the fluorophore is monitored. When the peptide is bound to MHC, there is a slower rotation leading to high polarisation, whereas when the peptide is free in solution, it rotates more rapidly, resulting in low polarisation [33]. In a competition‐based fluorescence polarisation assay, titration of a non‐fluorescent test peptide against a constant concentration of fluorescent peptide yields an IC_50_ value (the half‐maximum inhibitory concentration) for the test peptide of interest. FP assays measuring peptide binding to classical MHC class Ia proteins often use purified MHC class I proteins loaded with an easily exchangeable peptide, which can be either photocleavable [34], chemically cleavable [35], or a dipeptide [36]. Compared with classical MHC class Ia, both HLA‐E and Mamu‐E are known to refold with β2m in the absence of added peptide, and remain peptide‐receptive [27], enabling these proteins to be exploited in FP peptide exchange experiments without the requirement to produce pre‐loaded material with an exchangeable peptide.

In this paper, we outline the development of a nDSF‐based assay, alongside an FP‐based peptide competition approach, to gauge peptide binding to MHC‐E proteins, with a specific focus on HLA‐E*01:03 and Mamu‐E*02:04 subtypes. This combined approach allows the evaluation of peptide binding at scale and provides an approach in which small T_m_ changes correspond to log‐scale IC_50_ values, thus providing sensitive and relative quantitative measures of peptide binding to MHC‐E.

## 2 Results

### 2.1 Nanodifferential Scanning Fluorimetry Can be Applied to MHC‐E‐Based Thermal Stability Profiling

**FIGURE 1:**
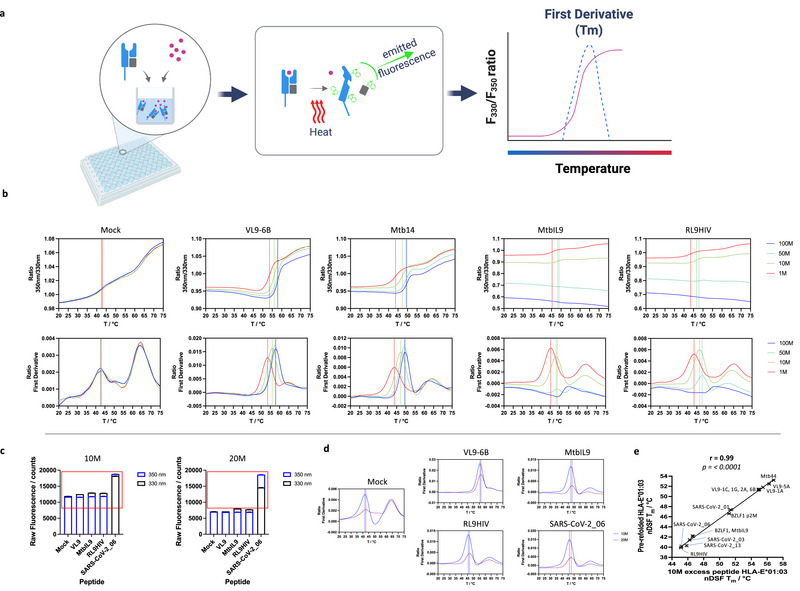
nano‐Differential scanning fluorimetry (nDSF) MHC‐E thermal stability assay optimisation. (a) A schematic demonstrating the nDSF MHC‐E thermal stability assay. The test peptide is first incubated with peptide “empty” MHC‐E and then subjected to a thermal ramp while fluorescence at 330 and 350 nm is measured following the exposure of tryptophan side chains as the MHC complex unfolds. The 350 nm/330 nm ratio is plotted (pink line), and the peak of its first derivative—the point at which 50% of the complex is unfolded (blue dashed line)—indicates the test peptide sample's melting temperature (T_m_). (b) nDSF traces illustrate 350 nm/330 nm ratios (top) and first derivatives (bottom) over the course of the thermal ramp for test samples. Peptide “empty” HLA‐E*01:03 incubated with mock, VL9‐6B, MtbIL9, Mtb14, and RL9HIV at concentrations of 100 (blue), 50 (green), 10 (yellow), and 1 M (red) excess peptide. The melting temperature (T_m_) measured is indicated (vertical line) with the corresponding colour denoting peptide concentration. (c) The initial fluorescence measured for the HLA‐E*01:03 samples at 10 and 20 M excess peptide, including a tryptophan‐containing peptide SARS‐CoV‐2_06, is shown. The red box indicates the optimal fluorescence window for nDSF‐based data collection. (d) Comparisons of nDSF first derivatives traces comparing 10 M (blue) and 20 M (purple) excess peptide conditions for the tryptophan‐containing SARS‐CoV‐2_06 peptide when incubated with peptide “empty” HLA‐E*01:03. (e) A comparison of nDSF T_m_ measurements for “peptide” empty HLA‐E*01:03 when incubated with 10 M excess test peptide versus HLA‐E*01:03 purified away from excess peptide by size‐exclusion chromatography following in vitro refolding (pre‐refolded material). Error bars indicate the standard deviation between experimental runs for three technical repeats.

HLA‐E and Mamu‐E heavy chains, truncated close to the transmembrane region for in vitro refolding [27], contain nine tryptophan residues, plus human and rhesus β2‐microglobulin, each with two tryptophan residues, enabling the thermal stability of both MHC‐E complexes to be studied by nDSF. HLA‐E*01:03 represents one of the two most common human MHC‐E types globally, the second being HLA‐E*01:01 [37]. Mamu‐E*02:04 is a common rhesus MHC‐E subtype [38]. These MHC‐E types were specifically chosen as we have previously worked extensively on these proteins and have amassed a strong understanding of their biochemistries. We also have prior knowledge of their peptide binding repertoires using either the SCT‐ or ELISA‐based peptide binding assays [11, 13, 25]. Finally, both HLA‐E*01:03 and Mamu‐E*02:04 can be refolded and purified in the absence of added peptide to produce peptide “empty” MHC‐E‐β2m complexes [39]. This feature makes them readily amenable to this work [27], as these peptide “empty” MHC‐E complexes are receptive to peptide loading when subsequently incubated with individual peptides. We used this quality to generate a higher‐throughput, rapid, nDSF‐based peptide binding screening method (Figure 1A).

nDSF assays were carried out in Tris‐buffered saline since this buffer has traditionally been used for MHC class I and HLA‐E purification, and thermal stability measurements [11, 30, 40]. Using a defined concentration of HLA‐E based on prior nDSF experiments [11, 29, 30], we initially incubated “empty” HLA‐E*01:03 with a panel of test peptides at 100, 50, 10, and 1 molar (M) excess concentrations (Figure 1B). These experiments were performed in Tris pH 7, 150 mM NaCl buffer, as our preliminary screens suggest that HLA‐E*01:03 shows greater stability at this pH compared with the more alkaline conditions commonly used for the purification of classical MHC I‐peptide complexes [41] (Figure S1A, Table S1). The test peptides included previously studied HLA‐E epitopes: MHC Ia leader sequence VL9‐6B (HLA‐B*08:01_3–11_ – VMAPRTVLL), Mycobacteria (Mtb)‐derived peptides including MtbIL9 (Mtb EsxH_4–12_ – IMYNYPAML) and Mtb14 (Mtb Rv2932_483–491_ – RMAATAQVL), and the human immunodeficiency virus (HIV) derived RL9HIV peptide (HIV‐1 Gag_275–283_ – RMYSPTSIL) [25]. A mock negative control comprising buffer with the equivalent concentration of dimethyl sulfoxide (DMSO) was also included. The mock samples showed minimal difference in T_m_ across the four different concentrations, and the nDSF 350 nm/330 nm ratio and first derivative traces closely overlapped with each other, revealing a T_m_ of ∼42°C (Figure 1B). For VL9‐6B and Mtb14 peptides, the nDSF traces indicate consistent peptide concentration‐dependent increases in T_m_ compared with the mock sample, reaching ∼58°C or ∼50°C, respectively (Figure 1B). However, for MtbIL9 and RL9HIV, the traces were atypical in that the changes in the 350/330 nm ratio were inconsistent across the peptide concentrations, and there was no clear transition point at the highest peptide concentrations, such that the T_m_ value was undetermined at 100 M excess (Figure 1B). Analysis of the peptide sequences suggested this anomaly may be due to tyrosine residues in MtbIL9 and RL9HIV peptides that impact fluorescence at 330 nm (but not at 350 nm) due to their fluorescence spectra, hence increasing background fluorescence on nDSF traces at higher peptide concentration (Figure S1B). In a subsequent nDSF assay, where the RL9HIV peptide position 3 tyrosine was substituted with a proline residue, this confirmed that the problem was specific to the tyrosine residue when the peptide was tested at higher concentrations (Figure S1C).

We next questioned if the presence of a tryptophan residue in test peptides would also produce interference at 330 and 350 nm. Given that 50 and 100 M excess peptide concentrations were problematic for tyrosine‐containing peptides, we focused on lower—10 or 20 M—excess peptide only, and incubated empty HLA‐E*01:03 with the tryptophan‐containing peptide SARS‐CoV‐2_06 (SARS‐CoV‐2 ORF1ab_6109–6117_ – VLWAHGFEL) [11] alongside VL9‐6B, MtbIL9, and RL9HIV for comparison at these concentrations. The initial fluorescence discovery scan demonstrated that at 10 M excess peptide, all sample fluorescence fell within the ideal window for nDSF analysis. By contrast, with an excess of 20 M, the higher fluorescence at 330 and 350 nm of the tryptophan‐containing peptide had to be accommodated using a lower excitation power, and this resulted in a lower initial reading for the other samples containing non‐tryptophan peptides (Figure 1C), plus lower quality nDSF traces compared with 10 M peptide incubations (Figure 1D; Figure S1D). These data suggested that 10 M excess peptide prevented issues related to tryptophan fluorescence to produce reliable nDSF‐based data. Comparing the nDSF T_m_ measurements between the different concentrations of peptide (Table S2) indicated that 10 M excess represented the most reliable concentration, yielding a higher T_m_ for binding peptide over the background/mock sample without compromising the quality of the data.

Finally, we investigated how well the nDSF assay, with 10 M excess peptide added to peptide‐receptive MHC‐E, compared with MHC‐E previously refolded and purified away from excess peptide (referred to here as pre‐refolded material). We performed this test using HLA‐E*01:03 and a peptide panel for which we had, except for one peptide, previously accrued binding data [25]. These included all VL9 peptides [30] (Table S3), Mtb44 (Mtb Rv1484_53–61_ – RLPAKAPLL), MtbIL9, BZLF1 (Epstein‐Barr virus (EBV) BZLF1_39–47_ – SQAPLPCVL) and a version with methionine at position 2 (introduced as a preferred anchor to improve binding in the B pocket), RL9HIV, SARS‐CoV‐2_01 (SARS‐CoV‐2 ORF1ab_5556–5564_ – VMPLSAPTL), SARS‐CoV‐2_03 (SARS‐CoV‐2 Spike_269–277_ – YLQPRTFLL), SARS‐CoV‐2_06, and SARS‐CoV‐2_13 (SARS‐CoV‐2 ORF1ab_5315–5323_ – AMYTPHTVL). A strong correlation (Pearson's coefficient = 0.99) was observed (Figure 1E), indicating that the chosen conditions of 10 M excess peptide accurately reflect the hierarchy of pre‐refolded MHC‐E complex and, as such, provides validation for assay set‐up using these conditions.

### 2.2 nDSF MHC‐E Thermal Stability Testing Identifies Subtle Peptide Binding Differences Between HLA‐E*01:03 and Mamu‐E*02:04

**FIGURE 2:**
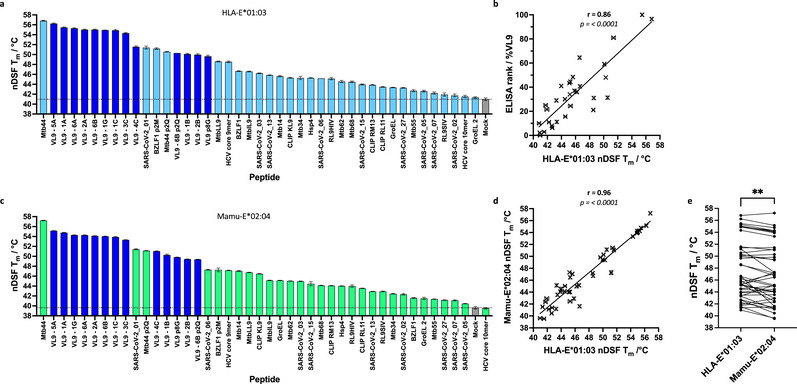
nDSF MHC‐E thermal stability assay, peptide screening, and assay comparison. (a) Test peptides incubated at 10 M excess with peptide “empty” HLA‐E*01:03 produce a range of T_m_ values as measured by nDSF. VL9 peptides are indicated in blue, and the mock (no added peptide) sample is indicated in grey. (b) A plot of HLA‐E*01:03 peptide binding data acquired by nDSF T_m_ versus ELISA rank data previously acquired using a peptide exchange sandwich ELISA binding assay is shown. (c) Test peptides incubated at 10 M excess with peptide “empty” Mamu‐E*02:04 at 10 M excess assay produced a range of T_m_ values as measured by nDSF. VL9 peptides are indicated in blue, and the mock sample is indicated in grey. (d, e) Strong correlation of nDSF T_m_ value acquired for HLA‐E*01:03 and Mamu‐E*02:04 is noted. Error bars in (a–d) represent the standard deviation between experimental runs for three technical repeats.

We next screened a panel of 43 HLA‐E/Mamu‐E binding peptides reported in previous studies (Table S3) [8, 9, 10, 11, 12, 13, 25, 26, 32, 40, 41, 42, 43] at 10 M excess peptide. For HLA‐E*01:03 (Figure 2A), a broad range of measured T_m_ values was obtained, from as high as 56.8°C for Mtb44 to 41.3°C for GroEL2 (Salmonella GroEL_287–295_ – AMLQDIATL), which measured only slightly above the mock control (41.0°C) (Figure 2A). Fifteen peptides produced T_m_ values above 50°C, which mostly comprised VL9 peptides; 14 T_m_ values ranged from 50°C to 45°C, and 14 fell between 45°C and the mock control T_m_. The latter two groups primarily comprised pathogen‐derived peptides. We observed a strong correlation (Pearson's coefficient = 0.86) between our nDSF recorded T_m_ values and previously reported measurements from HLA‐E peptide exchange binding assays [11, 25], suggesting strong concordance between both assays (Figure 2B).

We then performed nDSF assays for Mamu‐E*02:04 using the same peptide panel as tested for HLA‐E*01:03. A similar range of T_m_ values was recorded (Figure 2C). All peptides produced a T_m_ above the mock sample except for the HCV Core 10mer peptide (HCV Core_35–44_ – YLLPRRGPRL). Similar to HLA‐E*01:03, Mtb44 produced the highest T_m_ at 57.2°C for Mamu‐E*02:04 but in contrast, SARS‐CoV‐2_05 (SARS‐CoV‐2 Spike_505–513_ – YQPYRVVVL) produced the lowest T_m_ at 40.5°C, whereas GroEL2 yielded a T_m_ of 41.5°C compared with the mock sample (39.6°C) (Figure S2A). 13 peptides produced T_m_ values above 50°C, which like HLA‐E*01:03, mostly comprised VL9 peptides, 12 T_m_ values fell between 50°C and 45°C, and 17 measured between 45°C and the mock sample. Interestingly, the mock (no‐peptide) sample for Mamu‐E*02:04 measured a lower T_m_ compared with HLA‐E*01:03 (39.6°C vs. 41.0°C)—this is at odds with the yields of peptide “empty” MHC‐β2m refolds, which are consistently much greater for Mamu‐E*02:04. In terms of peptide binding, although there was a very strong correlation between HLA‐E*01:03 and Mamu‐E*02:04 T_m_ values (Pearson's coefficient = 0.96, Figure 2D), relative T_m_ values were lower for Mamu‐E*02:04 compared with HLA‐E*01:03 (Figure 2E). However, some deviation from the best fit curve was observed, with a few peptides resulting in higher T_m_ values for Mamu‐E*02:04 compared with HLA‐E*01:03 (e.g., Mtb14, RL9SIV (SIV Gag_277–285_ – RMYNPTNIL)) or a higher T_m_ for HLA‐E*01:03 compared with Mamu‐E*02:04 (e.g., BZLF1; Table S4, Figure S2A,B).

### 2.3 Fluorescence Polarisation Competition‐Based Assays Reveal Broad Differences in Relative Peptide Binding Strength

**FIGURE 3:**
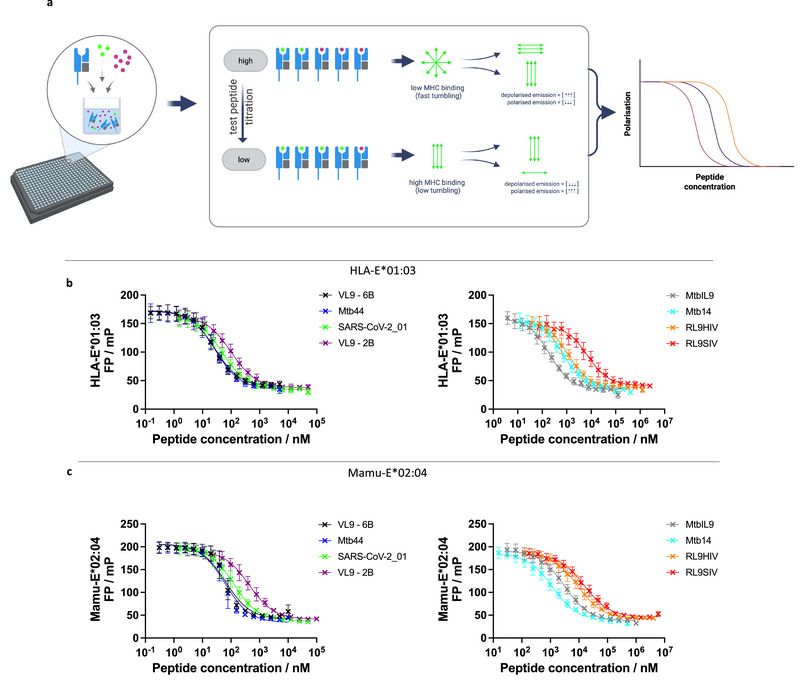
Fluorescence polarisation (FP) peptide competition assay for HLA‐E*01:03 and Mamu‐E*02:04. (a) A schematic demonstrating the FP peptide competition assay. A mix of test peptide and FITC‐labelled VL9 (VL9‐FITC) is incubated with empty MHC‐E. Polarised light excites VL9‐FITC, and emission polarisation is measured. At low test peptide, a higher concentration of FITC‐labelled peptide is bound by MHC‐E, resulting in slow rotation/ tumbling and high polarisation. At higher concentrations of test peptide, there is a greater concentration of non‐MHC‐E bound FITC‐labelled VL9 peptide, resulting in faster rotation/tumbling and low polarisation. A binding curve is generated based on the titration of the test peptide in competition with VL9‐FITC. (b, c) FP peptide competition assays where the test peptide was titrated tw‐ofold, with dilutions centred around the initial IC_50_ measurements of individual peptides, with a constant concentration of 100 nM VL9‐FITC peptide, run for HLA‐E*01:03 (b) and Mamu‐E*02:04 (c). FP data were fitted using [inhibitor] versus response (three parameters) non‐linear regression to calculate an IC_50_ value for a given test peptide. The titration curves represent the average of three independent FP competition assay repeat runs, with error bars indicating the standard deviation between FP assays.

We next investigated peptide binding to HLA‐E*01:03 and Mamu‐E*02:04 using fluorescence polarisation (FP)‐based peptide competition assays (Figure 3A). In these assays, MHC‐E was incubated with a titration of test peptide, which competed against a constant concentration of fluorescently labelled VL9 peptide (VL9‐FITC).

Before performing competition assays, we first confirmed binding of the VL9‐FITC peptide to HLA‐E*01:03 and Mamu‐E*02:04 by nDSF (Figure S3A). Using FP, we next determined that 100 nM of peptide “empty” HLA‐E*01:03 and Mamu‐E*02:04 was sufficient to achieve near‐maximum binding of 100 nM VL9‐FITC peptide (Figure S3B,F). To simultaneously check complex stability and measure the half‐life of VL9‐FITC‐loaded MHC‐E complexes, we loaded peptide “empty” HLA‐E*01:03 or Mamu‐E*02:04 proteins with VL9‐FITC peptide before the addition of either Dulbecco's phosphate‐buffered saline (DPBS) supplemented with DMSO (no peptide control), or 100 µM VL9‐6B peptide (Figure S3C,G). This assay showed that, with the addition of a competing peptide to block rebinding of VL9‐FITC peptide (100 µM VL9‐6B vs. 100 nM of VL9‐FITC peptide), the half‐lives of the VL9‐FITC‐HLA‐E*01:03 complex were approximately 1.5 h compared with approximately 0.6 h for the VL9‐FITC‐Mamu‐E*02:04 complex. By contrast, polarisation levels remained high and essentially unchanged over 2 days when buffer was added to the VL9‐FITC‐loaded MHC‐E complexes. This indicated that despite the relatively short half‐lives with which the VL9‐FITC peptide was bound, both MHC‐E molecules remained receptive to peptide loading throughout the test period of 2 days.

**FIGURE 4:**
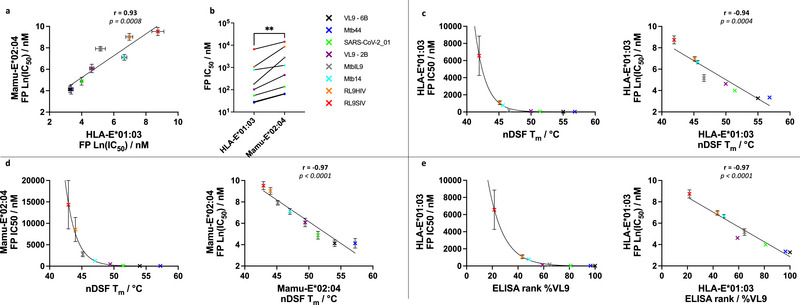
IC_50_ correlations for HLA‐E*01:03 and Mamu‐E*02:04 acquired data. (a) A comparison of HLA‐E*01:03 and Mamu‐E*02:04 Ln (IC_50_) values acquired by FP. (b) A side‐by‐side comparison illustrating the trajectory of FP‐Ln (IC_50_) for HLA‐E*01:03 and Mamu‐E*02:04 for the panel of test peptides is shown. (c, d) Exponential relationship between FP IC_50_ values measured for HLA‐E*01:03 (c) and Mamu‐E*02:04 (d) with corresponding nDSF T_m_ values measured for the corresponding peptides is shown with plots of the Ln (IC_50_) showing a strong correlation. (e) Exponential relationship between the HLA‐E*01:03 FP IC_50_ and ELISA rank data previously acquired using a sandwich ELISA peptide binding assay [11, 27] is shown with a plot of the Ln (IC_50_) showing a strong correlation.

We conducted FP peptide competition experiments with a small test panel of peptides that covered a range of low to high MHC‐E binders based on our nDSF results (Figure S2B). In these initial experiments, the competing test peptides were serially diluted (four‐fold) from 2 mM, over a range of 15 dilutions (Figure S3D,E,H,I). Subsequently, more focused FP peptide competition assays were performed in which competing peptides were 2‐fold serially diluted, with dilutions centred around the initial IC_50_ measurements of individual peptides (Figure 3B,C). The IC_50_ values obtained from these experiments revealed a 3‐log fold difference in IC_50_ values, ranging from low nM for peptides VL9‐6B and Mtb44, to µM for RL9HIV and RL9SIV peptides (Table 1; Figure 3). When compared separately, IC_50_ values for HLA‐E*01:03 and Mamu‐E*02:04 correlated with each other (Pearson's coefficient = 0.93, Figure 4A), although IC_50_ values were higher for Mamu‐E*02:04 compared with HLA‐E*01:03 (Figure 4B). The data also reflected the differences in peptide binding preferences previously observed by nDSF, including the HLA‐E*01:03‐specific binding preference for MtbIL9 compared with Mtb14, and the opposite pattern for Mamu‐E*02:04.

**TABLE 1:** Fluorescence polarisation (FP) based IC_50_ values measured for HLA‐E*01:03 and Mamu‐E*02:04 and test peptides.

Peptide	Sequence	HLA‐E*01:03 IC_50_	Mamu‐E*02:04 IC_50_
VL9 ‐ 6B	VMAPRTVLL	27 ± 3 nM	64 ± 18 nM
Mtb44	RLPAKAPLL	29 ± 4 nM	66 ± 30 nM
SARS‐CoV‐2_01	VMPLSAPTL	55 ± 4 nM	138 ± 51 nM
VL9 ‐ 2B	VTAPRTVLL	103 ± 15 nM	485 ± 179 nM
MtbIL9	IMYNYPAML	182 ± 51 nM	2.8 ± 0.6 µM
Mtb14	RMAATAQVL	782 ± 122 nM	1.2 ± 0.3 µM
RL9HIV	RMYSPTSIL	1.08 ± 0.22 µM	8.6 ± 2.8 µM
RL9SIV	RMYNPTNIL	6.6 ± 2.3 µM	14.3 ± 5.6 µM

*Note*: Fluorescence polarisation (FP) assay‐based half maximal inhibitory concentrations (IC_50_) ± standard deviation values for HLA‐E*01:03 and Mamu‐E*02:04 with a panel of test peptides are reported. Peptide IDs and corresponding sequences are shown.

We next directly evaluated the relationship between the FP‐based IC_50_ values and T_m_ values obtained by nDSF. The IC_50_ values appeared to follow an exponential relationship with nDSF T_m_ values, as shown by the strong linear relationship between Ln(IC_50_) and T_m_ for both HLA‐E*01:03 and Mamu‐E*02:04 (Pearson's coefficient = −0.94 and −0.97, respectively, Figure 4C,D). These results suggest that modest increments in T_m_ reflect substantial differences in IC_50_ values. We also evaluated the relationship between the IC_50_ values and previously published HLA‐E peptide exchange ELISA binding data [11, 25]. An exponential relationship was also observed between the IC_50_ values and % VL9 rank binding sandwich ELISA assay data, with a subsequent plot of the Ln (IC_50_) against % VL9 rank yielding a strong linear correlation (Pearson's coefficient = −0.97, Figure 4E). This again reflects that small binding differences measured by ELISA reflect significant IC_50_ shifts, and hence substantial relative binding strength differences.

## 3 Discussion

In this study, we developed a nDSF thermal stability‐based assay that provides a rapid and simplified platform to assess peptide binding to MHC‐E. When combined with an FP‐based peptide competition assay, detailed measures of relative peptide binding strengths were obtained that provide a more accurate gauge of differences, particularly for low‐affinity binding peptides, that heretofore were not fully recognised.

Renewed interest in MHC‐E as a T cell restriction element has driven the development of various peptide‐binding assays, and although several are robust, there is a requirement for the development of improved and nuanced peptide‐binding methodologies. The SCT cell surface expression assay is constructed so that the test peptide is linked to the β_2_m; however, as this peptide is free to dissociate and re‐bind, this might overestimate peptide binding strength, particularly for weaker binding peptides, which have shorter half‐lives when physiologically presented as non‐linked peptides on HLA‐E at the cell surface [44, 45]. For Mamu‐E, it has also been reported that the MHC‐E‐specific 4D12 antibody binds to peptide‐empty molecules, limiting the distinction of peptide‐loaded from empty/unloaded molecules [46]. Although the peptide‐exchange sandwich ELISA samples non‐linked MHC‐E‐peptide complexes and is therefore more physiological, especially since the stability of the complex is assessed following removal from peptide, the assay relies on several reagents, making it highly dependent on reagent quality and batch variation, resulting in a degree of assay‐to‐assay variation which is more apparent for weaker binding peptides [39].

In this paper, our results show that nDSF acquired data using the Tris‐buffer system described here was highly reproducible, producing minimal assay‐to‐assay variation during repeated runs, even when using independently produced batches of HLA‐E*01:03 and Mamu‐E*02:04 proteins. As T_m_ values can be sensitive to changes in experimental conditions [47], reporting data contingent on buffer composition, pH, and protein concentration is imperative.

When nDSF data acquired for HLA‐E*01:03 and Mamu‐E*02:04 were compared with respective FP‐competition assay‐based IC_50_ data, an exponential relationship was observed for both. Crucially, these exponential relationships signify that small T_m_ increments reflect substantial IC_50_ differences, a finding that is particularly evident for weaker binding peptides. Due to the strong linear correlation between Ln (IC_50_) and T_m_, accurate estimations of IC_50_ values from T_m_ measurements can now be calculated reliably.

Our comparison of HLA‐E*01:03 with Mamu‐E*02:04 showed that both nDSF and FP IC_50_ datasets correlated strongly, suggesting their sharing of similar peptide repertoires. This is to be expected given their near‐identical PBGs [27]. However, data obtained by nDSF, and supported by FP (albeit the latter based on fewer peptides), suggested that subtle peptide preferences exist for HLA‐E*01:03 compared with Mamu‐E*02:04. Additionally, for all peptides, higher IC_50_ values were recorded for Mamu‐E*02:04, and in the FP dissociation assay, shorter half‐lives (and therefore faster dissociation rates) with the VL9‐FITC peptide compared with HLA‐E*01:03, suggesting stronger binding of the peptides tested here to HLA‐E*01:03 compared with Mamu‐E*02:04. The difference in peptide specificities is likely due to residue 73, the only non‐conserved PBG residue—isoleucine for HLA‐E*01:03, and threonine for Mamu‐E*02:04. For certain peptides, this could directly affect peptide binding at position 6, but also indirectly, via conformational effects impacting the peptide at positions 5 and 7. In HLA class Ia, residue 73 forms part of the C pocket, which, for certain MHC‐peptide complexes, can form interactions with positions 3 as well as positions 5/6 of the peptide mainchain [48]. Further peptide binding repertoire differences are likely for HLA‐E*01:03 compared with other Mamu‐E subtypes. These include Mamu‐E*02:30, which carries two additional changes at position 24 (serine to phenylalanine) and 74 (phenylalanine to leucine), which affect the B and C pockets, respectively, and also Mamu‐E*02:20, which shares the same changes at positions 24 and 74, but additionally carries a tyrosine to histidine change at position 99 in the D pocket [15, 27].

Other structural subtleties between these two MHC‐E molecules may play a role in overall peptide binding—these include residues that map to the α1 and α2 helices that could impact how MHC‐E binding pocket residues interact with the peptide side chains. These α‐helical changes could introduce some structural flexibility to Mamu‐E*02:04, which might be reflected in the higher yields of peptide “empty” Mamu‐E*02:04 following in vitro refolding, compared with peptide “empty” HLA‐E*01:03, despite its lower thermal stability [39]. However, the overall impact of these differences in relation to disparities in their peptide binding repertoire hierarchies is currently unknown and requires further exploration. Due to the absence of X‐ray crystal structures for Mamu‐E, the consequence of these differences currently remains unexplored.

Importantly, the peptides tested in this study were previously reported as HLA‐E binders, creating a bias to HLA‐E. Hence, there may exist peptides with binding preferences for Mamu‐E*02:04 over HLA‐E*01:03 that were not tested here. However, given the remarkable correlation of nDSF acquired data for HLA‐E*01:03 and Mamu‐E*02:04, a strong overall similarity in their peptide binding hierarchies is expected.

In summary, the nDSF assay described here reliably measured peptide binding to HLA‐E*01:03 and correlated strongly with nDSF measurements obtained using pre‐refolded HLA‐E*01:03 complexes and prior peptide binding sandwich ELISA data [11, 25]. The approach of combining nDSF and IC_50_ values obtained using a FP peptide competition assay allows for a more accurate and sensitive evaluation of relative peptide binding strength differences to be explored. In combination, these newer methodologies offer a platform that can be performed at scale, allowing the streamlined testing of candidate peptide epitopes to provide crucial MHC‐E binding information ahead of therapeutic target selection.

## 4 Data Limitation and Perspective

To better understand the extent of the peptide binding overlap and patterns where binding differences are noted between HLA‐E and Mamu‐E, larger panels of test peptides sourced in an unbiased manner should be explored. Additionally, testing of the various Mamu‐E subtypes with noted, albeit subtle, PBG differences would help tease apart specific differences in peptide binding repertoires.

## 5 Methods and Materials

### 5.1 Peptide Synthesis

Peptides were produced using Fmoc (9‐fluorenylmethoxy carbonyl) solid‐phase peptide synthesis and purified to >85% purity using HPLC (Genscript USA). Peptides were dissolved in DMSO at a 200 mM concentration and stored at −20°C.

### 5.2 MHC‐E Peptide Expression, Refolding and Purification

Human and Mamu β2‐microglobulin (β2m), and HLA‐E*01:03 and Mamu‐E*02:04 heavy chain residues 1–276 were expressed as inclusion bodies in *Escherichia coli* One Shot BL21 (DE3) pLysS competent bacterial cells (Invitrogen), and purified according to a previously described method [2, 27] involving sonication, homogenisation in a Triton‐based buffer, with final solubilisation in a 8 M urea‐MES pH 6.5 buffer. Solubilised proteins were dissolved at 2.0 mg/mL, aliquoted and stored at −80°C.

For MHC‐E protein refolds, a buffer consisting of 400 mM L‐arginine monohydrochloride, 5 mM reduced glutathione, 0.5 mM oxidised glutathione, 100 mM Tris pH 8.0 (all Sigma‐Aldrich), and 2 mM EDTA pH 8.0 (Invitrogen), in MilliQ water was equilibrated at 4°C for 30 min. β2m was added to a final concentration of 2 µM, and after 30 min, peptide was added to a final concentration of 30 µM, and MHC heavy chain was immediately pulsed into the refolding buffer to reach a final concentration of 1 µM. For peptide “empty” MHC, refolding was performed as above, but with the peptide omitted.

Following 72 h incubation at 4°C, refolded samples were filtered through a 1.0 µm cellular nitrate membrane (Cytiva) and concentrated using a VivaFlow 50R 10 kDa molecular weight cut‐off system (Sartorius), followed by a 10 kDa cut‐off Vivaspin Turbo Ultrafiltration centrifugal device (Sartorius). Subsequently, concentrated samples were separated by size exclusion chromatography using a Superdex S75 16/60 column (Cytiva) on a fast protein liquid chromatography (FPLC) AKTA Start System. Protein‐containing fractions were combined and concentrated to 2.0 mg/mL using a 10 kDa cut‐off Vivaspin Turbo Ultrafiltration centrifugal device (Sartorius). MHC‐E peptide complexes were stored in 20 mM Tris, pH 7.0, 150 mM NaCl buffer, whereas peptide “empty” MHC‐E were stored in 50 mM Tris, pH 7.0, 150 mM NaCl, 20% glycerol buffer. Sample aliquots were flash frozen in dry ice and stored at −80°C.

Previously reported HLA‐B*27:09 [49] and HLA‐A*24:02 [50] peptide complexes were produced analogously except with purification into 20 mM Tris pH 8.0, 150 mM NaCl buffer.

### 5.3 Nano‐Differential Scanning Fluorimetry MHC‐E Thermal Stability Assay

The thermal stability of test peptide‐loaded HLA‐E*01:03/Mamu‐E*02:04 was measured by nDSF. Nine micrograms of “empty” MHC‐E per reaction were incubated with a 10‐molar excess or otherwise specified concentration of test peptide in 50 mM Tris pH 7.0, 150 mM NaCl buffer, in a final volume of 20 µL, and incubated for 30 min at room temperature. DMSO concentration ranged from 0.5% to 0.05% (v/v). Individual samples were split into duplicates between two Prometheus Grade Standard Capillaries (Nanotemper) and placed into a Prometheus Panta (Nanotemper) instrument capillary tray. Excitation power was adjusted such that the initial fluorescence emission detection at wavelengths of 330 and 350 nm covered a range of 8000–15,000 raw fluorescence units, after which a thermal ramp at 1°C/min over a range of 20–95°C was applied. Automated thermal melt data calling was achieved using the Thermal Unfolding Analysis software within PR.Panta Analysis, v1.2. Duplicates were averaged, and experiments were repeated three times.

The thermal stability of HLA‐E*01:03 pre‐refolded with test peptide was measured using nDSF in an analogous way, at a concentration of 0.5 mg/ml in 20 mM Tris, pH 7.0, 150 mM NaCl. Thermal stability of HLA‐E*01:03, HLA‐B*27:09, and HLA‐A*24:02 pre‐refolded with peptide was assessed at the same concentration in 20 mM Tris pH 9.0, 8.0, or 7.0, and 150 mM NaCl.

### 5.4 Fluorescence Polarisation Peptide Competition Assay

Fluorescence polarisation (FP) experiments were used to evaluate the IC_50_ values of peptide binding in competition with a FITC‐labelled peptide. Experiments were all conducted in duplicate and with 60 µL reaction volumes in Dulbecco's phosphate‐buffered saline (DPBS; Sigma‐Aldrich), in a black 384‐well, non‐binding F‐bottom microplate (Greiner Bio‐One) at room temperature, using FITC‐labelled VL9 peptide VMAPK*TVLL (VL9‐FITC, where K* is lysine conjugated to a FITC fluorochrome), synthesised by Genscript USA, as described previously, at a final concentration of 100 nM. A PHERAstar FS plate reader (BMG LABTECH) with an FP 485 nm/520 nm/520 nm optic module was used to measure fluorescence polarisation.

Optimisation experiments involved titration of peptide “empty” HLA‐E*01:03 and Mamu‐E*02:04 from 800 nM over 16 concentrations (twofold serial dilution) with a constant concentration of 100 nM VL9‐FITC peptide. Fluorescence polarisation was measured after a 1 h incubation in the dark, and the data were plotted using Prism (Version 10.4.1, GraphPad) and fitted using [inhibitor] versus response (three parameters) non‐linear regression to calculate an IC_50_ value. This was used to calculate the 80%–90% VL9‐FITC peptide binding saturation window.

To measure the half‐life of the VL9‐FITC peptide bound to MHC‐E, peptide dissociation experiments were undertaken. Peptide “empty” HLA‐E*01:03 and Mamu‐E*02:04 were incubated overnight with VL9‐FITC peptide at room temperature in the dark, and subsequently, 100 µM VL9‐6B peptide or DPBS with the equivalent amount of DMSO was added, and fluorescence polarisation was measured immediately and then 1, 2, 3, 4, 8, 24, and 48 h later.

To measure IC_50_ values, 100 nM VL9‐FITC peptide was added to the non‐binding F‐bottom microplate in conjunction with the test peptide that was initially serially diluted fourfold from a top concentration of 2 mM over 15 concentrations. Peptide “empty” HLA‐E*01:03/Mamu‐E*02:04 was added, and fluorescence polarisation was measured after a 1 h incubation in the dark. Three subsequent repeats were carried out, but using a twofold serial dilution centred on the initial IC_50_ value measurement.

### 5.5 Analysis and Statistics

Data were analysed using Prism 10 (Version 10.4.1, GraphPad). Data points plotted show the mean ± standard deviation. Correlation graphs were fitted using standard linear regression, with a Pearson correlation coefficient and *p*‐value calculated. Dissociation and exponential decay curves were fitted using one‐phase decay non‐linear regression. A Wilcoxon signed‐rank test was used to compare the two groups. Titration curves were fitted using inhibitor versus response (three parameters) non‐linear regression.

## Author Contributions

Max N Quastel conceived the study, performed the experiments, analysed the data, and wrote the manuscript. Sashini A. Ranawana and Bas W.A. Peeters provided MHC‐E material and offered technical and scientific support. Andy van Hateren provided scientific guidance and overview of the FP‐based assays. Andrew J. McMichael provided an overview, supervision, and acquired funding for the project. Geraldine M. Gillespie conceived the study, helped write the manuscript, provided supervision, and acquired funding for the project. All authors helped review and provided edits for the final manuscript.

## Funding

We are grateful to the following funders for their support: The Wellcome Trust (227388/Z/23/Z), the Gates Foundation (INV‐055780), and the NIH (UM1 AI 164567‐02 and R01 AI 175459).

## Conflicts of Interest

M.N.Q., S.A.R., B.W.A.P., A.J.M., and G.M.G. are inventors on HLA‐E‐related patents owned by Oxford University Innovation. The remaining author declares no conflicts of interest.

## Supporting information




**Supporting File**: eji70125‐sup‐0001‐SuppMat.pdf.

## Data Availability

The data that support the findings of this study are available from the corresponding author upon reasonable request.
