# Supplementary material for: Shared HLA‐E and Mamu‐E Peptide Repertoires With Subtle Peptide Binding Differences Revealed by Combined nDSF‐ and Fluorescence Polarisation‐Based Methods
Source: Eur J Immunol. 2026 Jan 16;56(1):e70125. doi: 10.1002/eji.70125 (PMC12810216; doi:10.1002/eji.70125)
Supplement: Supplementary file 1 — Supporting File: eji70125‐sup‐0001‐SuppMat.pdf. [file EJI-56-e70125-s001.pdf]

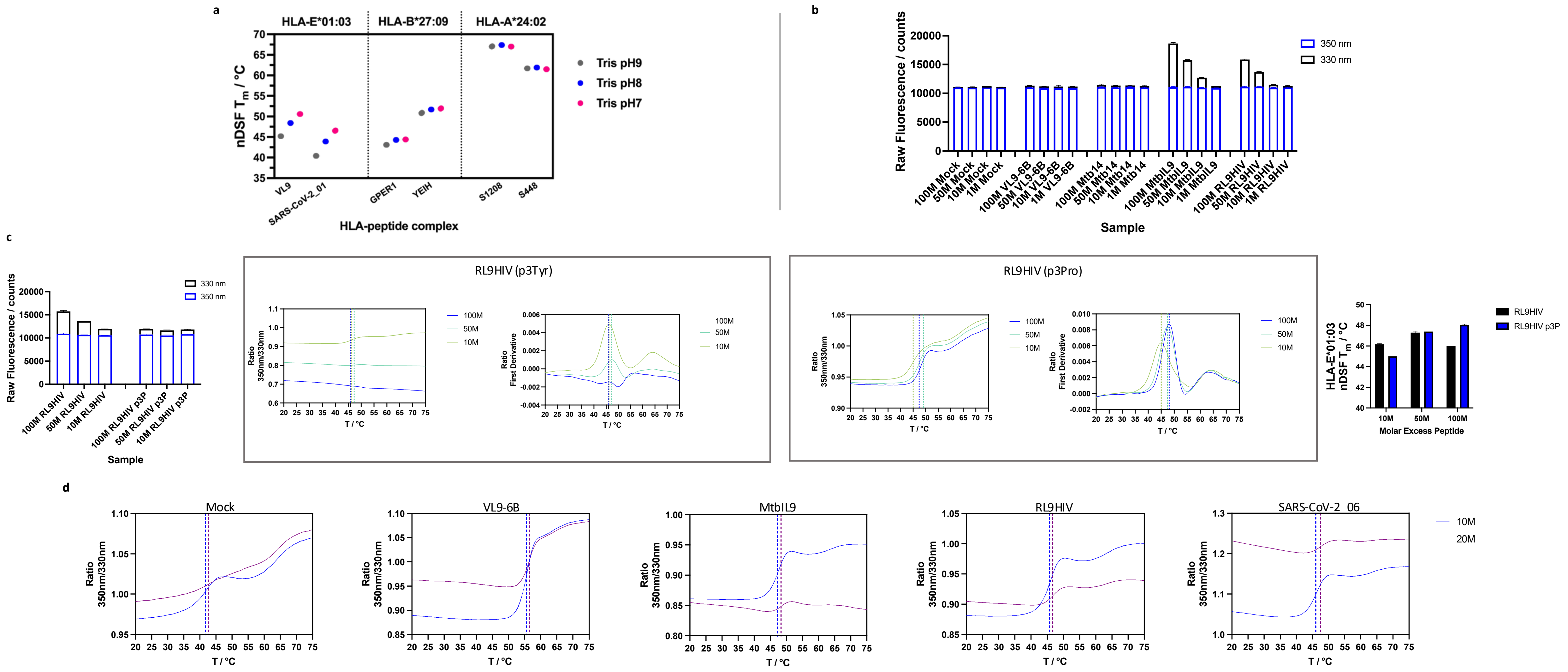

Figure 1S - nano-Differential scanning fluorimetry (nDSF) MHC-E. (a) nDSF thermal stability measurements of HLA-E*01:03, HLA-B27:09, and HLA-A24:02 peptide complexes in Tris buffer at pH 9, pH 8, and pH 7 **(b)** The initial fluorescence measured for the HLA-E*01:03 samples at 1-100M excess peptide for Mock, VL9-6B, Mtb14, MtbIL9, and RL9HIV peptide samples **(c)** Raw fluorescence counts, F350nm/F330nm ratio traces and first derivative T_m plots for HLA-E*01:03 loaded with 10, 50 and 100M of the index position 3 tyrosine containing RL9HIV peptide versus the position 3 proline substituted RL9HIV variant **(d)** Comparisons of 350nm/330nm ratio traces comparing the 10M (blue) and 20M (purple) excess peptide conditions when the Trp-containing SARS-CoV-2_06 peptide is included in the test samples.

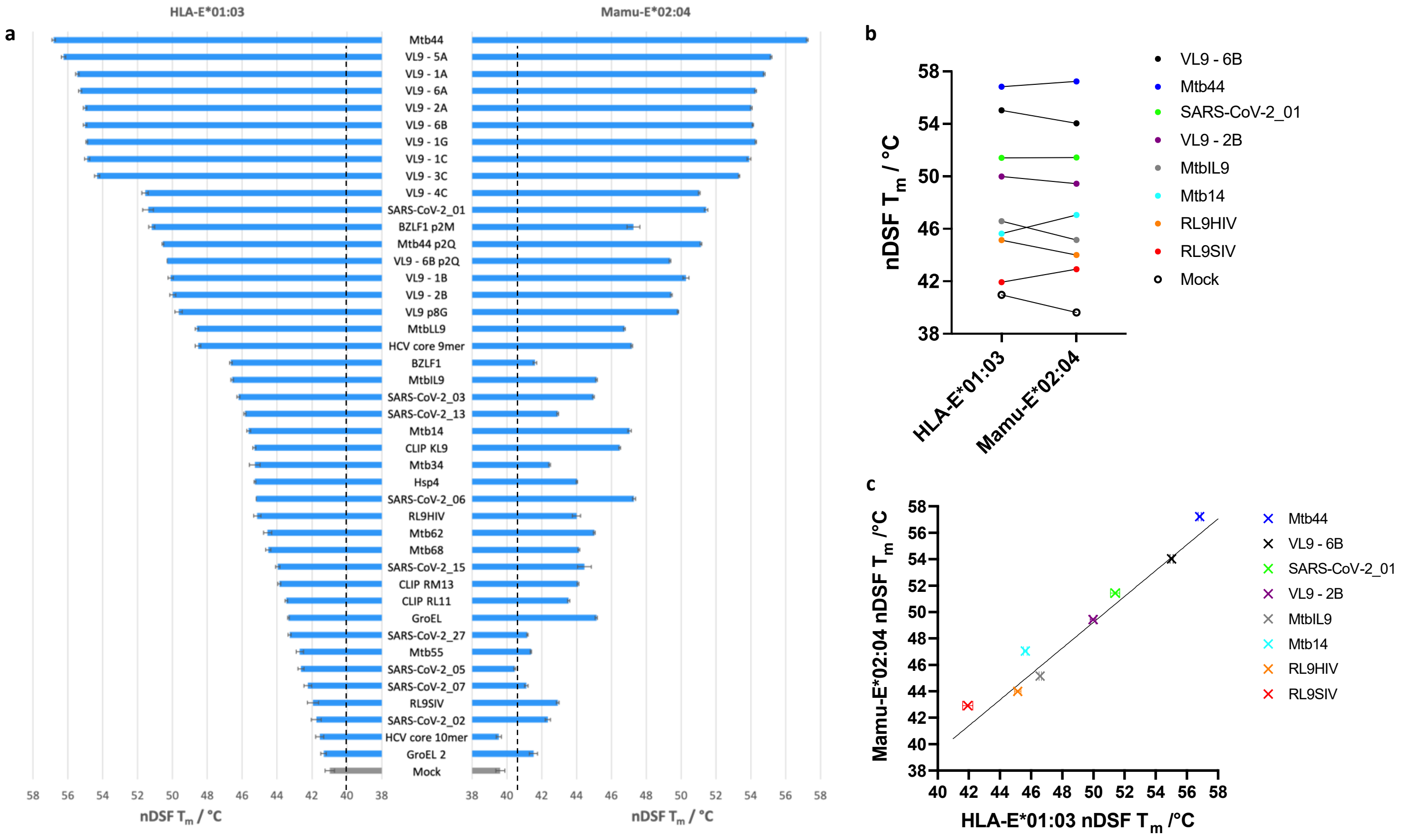

Figure 2S - **nDSF T_m comparison (a)** A comparison between HLA-E*01:03 and Mamu-E*02:04 nDSF T_m values with 10M excess peptide used **(b, c) P**lot comparing the nDSF T_m values for HLA-E*01:03 against Mamu-E*02:04 with 10M excess peptide, highlighting the peptides used in the fluorescent polarisation peptide competition assay. Error bars for **(a,c)** indicates the standard deviation between experimental runs.

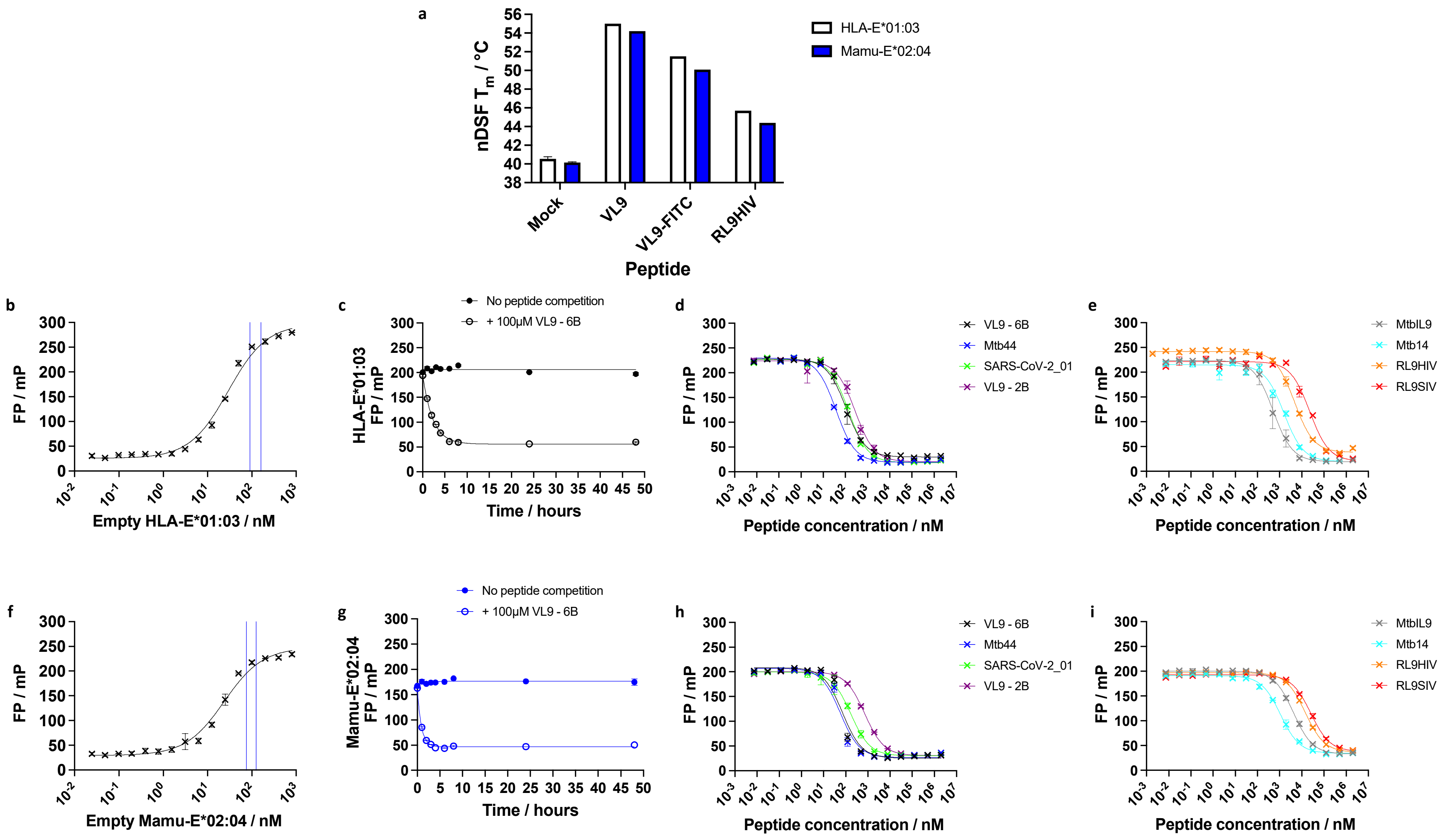

Figure 3S - Fluorescence polarisation peptide competition assay optimisation. (a) T_m values measured in the nDSF assay for both HLA-E*01:03 and Mamu-E*02:04 for the VL9-FITC peptide. **(b,f)** A plot of FP measured for peptide 'empty' HLA-E*01:03 **(b)** and Mamu-E*02:04 **(f)** titrated against a 100 nM of VL9-FITC peptide. 80-90% of VL9-FITC peptide binding is indicated between the two blue lines. **(c,g)** A FP dissociation experiment where HLA-E*01:03 (c) and Mamu-E*02:04 (g) were pre-incubated with 100 nM VL9-FITC peptide and then either no peptide competition (DPBS with equivalent DMSO) or 100 µM of VL9-6B peptide was added, and the FP monitored over time. **(d,e,h,i)** FP peptide competition assays where test peptide was titrated from 2 mM (4-fold) with a constant concentration of 100 nM VL9-FITC peptide and then added to HLA-E*01:03 **(d,e)** or Mamu-E*02:04 **(h,i).** The FP was measured and curve fitted to calculate an initial IC_{50} value for a given test peptide. Error bars in **(a-i)** indicate the standard deviation between replicates.

MHC allele	Peptide name	Sequence	Organism	Protein	Ref.	nDSF T_m / °C		
						Tris pH 7	Tris pH 8	Tris pH9
HLA-E*01:03	VL9-6B	VMAPRTVLL	Human	HLA-B*07:02$_{3-11}$	Lin, Z. *et al.* (2023)[32]	45.2	48.4	50.6
	SARS-CoV-2_01	VMPLSAPTL	SARS-CoV-2	ORF1ab$_{5556-5564}$	Yang, H. *et al.* (2023)[11]	40.4	43.9	46.5
HLA-B*27:09	GPER1	GQMWLLAPR	Human	GPER1$_{314-322}$	Yang, X. *et al.* (2023)[45]	43.1	44.3	44.4
	YEIH	LRVMMLAPF	*E. coli*	YEIH$_{232-240}$		50.8	51.7	51.9
HLA-A*24:02	S1208	QYIKWPWYI	SARS-CoV-2	Spike$_{1208-1216}$	Rowntree, L. *et al.* (2021)[46]	67.0	67.4	67.0
	S448	NYNYLYRLF	SARS-CoV-2	Spike$_{448-456}$		61.7	61.9	61.5

Table S1. HLA-E*01:03, HLA-B*27:05 and HLA-B*24:02 peptide complexes tested by nDSF

SARS-CoV-2 – Severe acute respiratory syndrome coronavirus 2; *E.coli* – *Escherichia coli*

Molar excess of peptide	Mock	VL9-6B	MtbIL9	Mtb14	RL9HIV
100	42.6 °C	57.9 °C	n.d.	49.5 °C	n.d.
50	42.7 °C	57.6 °C	48.9 °C	49.2 °C	47.8 °C
10	42.7 °C	56.0 °C	48.2 °C	47.3 °C	46.5 °C
1	42.9 °C	53.6 °C	45.3 °C	43.7 °C	43.5 °C

Table S2. nDSF Tm values measured for 'empty' HLA-E*01:03 incubated with molar excess peptide.
n.d.- not determined.

Peptide name	Sequence	Amino acids	Organism	Protein	Ref.
VL9-1A	VMAPRTLLL	9	Human	HLA-A*01:01$_{3-11}$	Lin, Z. *et al.* (2023)[32]
VL9-1B	VTAPRTLLL	9	Human	HLA-B*13:01$_{3-11}$	
VL9-2A	VMAPRTLVL	9	Human	HLA-A*02:01$_{3-11}$	
VL9-2B	VTAPRTVLL	9	Human	HLA-B*15:01$_{3-11}$	
VL9-3C	VMAPRALLL	9	Human	HLA-C*07:01$_{3-11}$	
VL9-4C	VMAPQALLL	9	Human	HLA-C*17:01$_{3-11}$	
VL9-5A	VMPPRTLLL	9	Human	HLA-A*80:01$_{3-11}$	
VL9-6B	VMAPRTVLL	9	Human	HLA-B*07:02$_{3-11}$	
VL9-1C	VMAPRTLIL	9	Human	HLA-C*01:02$_{3-11}$	
VL9-1G	VMAPRTLFL	9	Human	HLA-G*01:01$_{3-11}$	
VL9-6A	IMAPRTLVL	9	Human	HLA-A*34:01$_{3-11}$	
VL9 p8G	VMAPRTLGL	9	Human	HLA-A*01:01$_{3-11}$ p8G	
VL9-6B p2Q	VQAPRTVLL	9	Human	HLA-B*07:02$_{3-11}$ p2Q	
Hsp4	QMRPVSRVL	9	Human	Hsp60sp$_{10-18}$	Michaëlsson, J. *et al.* (2002)[42]
CLIP KL9	KMRMATPLL	9	Human	CD74$_{106-114}$	In-lab
CLIP RL11	RMATPLLMQAL	11	Human	CD74$_{108-118}$	
CLIP RM13	RMATPLLMQALPM	13	Human	CD74$_{108-120}$	
Mtb44	RLPAKAPLL	9	Mtb	Rv1484$_{53-61}$	Joosten, S. A. *et al.* (2010)[12]
Mtb44 p2Q	RQPAKAPLL	9	Mtb	Rv1484$_{53-61}$ p2Q	
Mtb14	RMAATAQVL	9	Mtb	Rv2932$_{483-491}$	
Mtb34	VMTTVLATL	9	Mtb	Rv1734c$_{42-50}$	
Mtb55	VMATRRNVL	9	Mtb	Rv1518$_{240-248}$	
Mtb62	RMPPLGHEL	9	Mtb	Rv2997$_{470-478}$	
Mtb68	VLRPGGHFL	9	Mtb	Rv1523$_{251-259}$	
MtbIL9	IMYNYPAML	9	Mtb	EsxH$_{4-12}$	McMurtrey, C. *et al.* (2017)[9]
MtbLL9	LLDAHIPQL	9	Mtb	EsxG$_{3-11}$	
GroEL	KMLRGVNVL	9	Salmonella	GroEL$_{15-23}$	Salerno-Gonçalves, R. *et al.* (2004)[8]
GroEL2	AMLQDIATL	9	Salmonella	GroEL$_{287-295}$	
SARS-CoV-2_01	VMPLSAPTL	9	SARS-CoV-2	ORF1ab$_{5556-5564}$	Yang, H. *et al.* (2023)[11]
SARS-CoV-2_02	VMYASAVVL	9	SARS-CoV-2	ORF1ab$_{3683-3691}$	
SARS-CoV-2_03	YLQPRTFLL	9	SARS-CoV-2	Spike$_{269-277}$	
SARS-CoV-2_05	YQPYRVVVL	9	SARS-CoV-2	Spike$_{505-513}$	
SARS-CoV-2_06	VLWAHGFEL	9	SARS-CoV-2	ORF1ab$_{6109-6117}$	
SARS-CoV-2_07	YMPYFFTLL	9	SARS-CoV-2	ORF1ab$_{2168-2176}$	
SARS-CoV-2_13	AMYTPHTVL	9	SARS-CoV-2	ORF1ab$_{5315-5323}$	
SARS-CoV-2_15	SLPINVIVF	9	SARS-CoV-2	ORF1ab$_{2535-2543}$	
SARS-CoV-2_27	QLPAPRTLL	9	SARS-CoV-2	ORF1ab$_{5728-5736}$	
BZLF1	SQAPLPCVL	9	EBV	BZLF1$_{39-47}$	Jørgensen, P. B. *et al.* (2012)[10]
BZLF1 p2M	SMAPLPCVL	9	EBV	BZLF1$_{39-47}$ p2M	
RL9HIV	RMYSPTSIL	9	HIV-1	Gag$_{275-283}$	Walters, L. C. *et al.* (2018)[27]

Table S3. Peptides tested by nDSF for HLA-E*01:03 and MamuE*02:04

Mtb – *Mycobacteria tuberculosis*; *Salmonella* – *Salmonella enterica serovar Typhi*; SARS-CoV-2 – Severe acute respiratory syndrome coronavirus; EBV – Epstein Barr Virus; HIV-1 – Human Immunodeficiency Virus 1; SIV – Simian Immunodeficiency Virus; HCV - Hepatitis C Virus

Peptide	HLA-E*01:03		Mamu-E*02:04	
	nDSF T_m / °C	SD	nDSF T_m / °C	SD
Mtb44	56.82	0.10	57.23	0.05
VL9-5A	56.25	0.12	55.15	0.05
VL9-1A	55.47	0.10	54.77	0.05
VL9-6A	55.3	0.09	54.27	0.05
VL9-2A	55.02	0.10	54.12	0.04
VL9-6B	55.02	0.10	54.03	0.05
VL9-1G	54.92	0.04	54.28	0.04
VL9-1C	54.9	0.15	53.9	0.11
VL9-3C	54.33	0.14	53.32	0.04
VL9-4C	51.57	0.18	51.05	0.05
SARS-CoV-2_01	51.4	0.30	51.43	0.10
BZLF1 p2M	51.2	0.17	47.27	0.37
Mtb44 p2Q	50.55	0.05	51.13	0.05
VL9-6B p2Q	50.3	0.00	49.37	0.05
VL9-1B	50.1	0.15	50.28	0.17
VL9-2B	49.98	0.18	49.43	0.05
VL9 p8G	49.65	0.21	49.82	0.04
MtbLL9	48.6	0.09	46.75	0.05
HCV core 9mer	48.53	0.16	47.17	0.05
BZLF1	46.65	0.08	41.62	0.10
MtbIL9	46.58	0.08	45.15	0.05
SARS-CoV-2_03	46.22	0.08	44.97	0.05
SARS-CoV-2_13	45.85	0.05	42.92	0.04
Mtb14	45.63	0.12	47.05	0.10
CLIP KL9	45.3	0.09	46.47	0.05
Mtb34	45.28	0.31	42.45	0.05
Hsp4	45.27	0.05	44.02	0.04
SARS-CoV-2_06	45.2	0.00	47.3	0.09
RL9HIV	45.14	0.21	44	0.24
Mtb62	44.55	0.23	45.02	0.04
Mtb68	44.5	0.15	44.13	0.05
SARS-CoV-2_15	43.97	0.12	44.45	0.40
CLIP RM13	43.87	0.08	44.07	0.05
CLIP RL11	43.47	0.08	43.55	0.05
GroEL	43.35	0.05	45.13	0.05
SARS-CoV-2_27	43.28	0.08	41.18	0.04
Mtb55	42.7	0.21	41.38	0.04
SARS-CoV-2_05	42.63	0.18	40.45	0.05
SARS-CoV-2_07	42.23	0.22	41.12	0.10
RL9SIV	41.93	0.32	42.92	0.08
SARS-CoV-2_02	41.75	0.28	42.35	0.15
HCV core 10mer	41.55	0.24	39.53	0.15
GroEL2	41.33	0.16	41.53	0.23
Mock	40.97	0.27	39.62	0.26

Table S4. nDSF T_m values measured for 'empty' HLA-E*01:03 incubated with 10M excess peptide.
n.d. - not determined. SD - standard deviation between experimental runs
